# PPP1R14B is a diagnostic prognostic marker in patients with uterine corpus endometrial carcinoma

**DOI:** 10.1111/jcmm.17697

**Published:** 2023-02-23

**Authors:** Kang He, Taiwei Wang, Xuemiao Huang, Zhaoyun Yang, Zeyu Wang, Shuang Zhang, Xin Sui, Junjie Jiang, Lijing Zhao

**Affiliations:** ^1^ Department of Rehabilitation, School of Nursing Jilin University Changchun China; ^2^ Department of Rehabilitation The Second Hospital of Jilin University Changchun China

**Keywords:** bioinformatics, nomogram, PPP1R14B, prognosis, uterine corpus endometrial carcinoma

## Abstract

Uterine corpus endometrial carcinoma (UCEC) is one of the most common malignancies of the female genital tract. A recently discovered protein‐coding gene, PPP1R14B, can inhibit protein phosphatase 1 (PP1) as well as different PP1 holoenzymes, which are important proteins regulating cell growth, the cell cycle, and apoptosis. However, the association between PPP1R14B expression and UCEC remains undefined. The expression profiles of PPP1R14B in multiple cancers were analysed based on TCGA and GTE databases. Then, PPP1R14B expression in UCEC was investigated by gene differential analysis and single gene correlation analysis. In addition, we performed gene ontology term analysis, Kyoto Encyclopedia of Genes and Genomes pathway analysis, gene set enrichment analysis, and Kaplan–Meier survival analysis to predict the potential function of PPP1R14B and its role in the prognosis of UCEC patients. Then, a tool for predicting the prognosis of UCEC, namely, a nomogram model, was constructed. PPP1R14B expression was higher in UCEC tumour tissues than in normal tissues. The results revealed that PPP1R14B expression was indeed closely associated with tumour development. The results of Kaplan–Meier plotter data indicated that patients with high PPP1R14b expression had poorer overall survival, disease‐specific survival, and progression‐free interval than those with low expression. A nomogram based on the results of multifactor Cox regression was generated. PPP1R14B is a key player in UCEC progression, is associated with a range of adverse outcomes, and can serve as a prognostic marker in the clinic.

## 1 INTRODUCTION

Uterine corpus endometrial carcinoma (UCEC) is one of the most common malignancies in the female genital tract, with over 200,000 new cases diagnosed worldwide each year.^1^, ^2^ In recent years, the morbidity and mortality of UCEC have been increasing over time due to the increase in life expectancy of the population and the overall prevalence of obesity and metabolic syndrome, and the age of onset has been decreasing, posing a serious threat to women's health and lives.^3^, ^4^, ^5^ Although we have made great progress in the treatment of UCEC in recent years, there is still a dearth of viable therapeutic options for advanced recurrent UCEC.^6^ As a result, discovering and identifying new compounds that may be used as prognostic biomarkers and therapeutic targets in the treatment of UCEC is critical.

The PPP1R14B gene, also known as PLCB3N, SOM172, or PNG, is the protein‐encoding gene located on chromosome region 11q13 close to the human phosphatidylinositol‐specific phospholipase Cβ3 gene (PLCB3).^7^ The PPP1R14B protein is capable of inhibiting protein phosphatase 1 (PP1) as well as different PP1 holoenzymes.^8^, ^9^ PP1, a widespread Ser‐/Thr‐specific phosphatase in organisms, plays a key role in numerous biological processes, such as RNA splicing, protein synthesis, control of cell cycle progression, promotion of apoptosis, and glycogen metabolism.^10^, ^11^, ^12^ These biological processes are critical in the development of tumorigenesis and influence numerous functions, such as tumour growth, invasion, and metastasis. Studies have shown that PP1 cannot only inhibit the mitosis of tumour cells but also promote cell apoptosis when the cells are damaged beyond repair.^11^ Upregulation of PPP1R14B inhibits the expression of protein phosphatase 1 (PP1)^13^ and inhibits the function of PP1 to regulate cell growth and the cell cycle and promote apoptosis by inhibiting the myosin, glycogen‐related holoenzyme, and monomeric catalytic subunits of PP1.^8^ These effects may further lead to the proliferation, metastasis, and invasion of tumour cells.

A previous study found that PPP1R14B was significantly overexpressed in ovarian clear cell carcinoma (OCCC) and endometriosis.^14^ Another study found that the mRNA expression of PPP1R14B was significantly higher in the plasma of patients with prostate cancer.^15^ A recent study also showed that PPP1R14B was highly expressed in tumour tissues, and its high expression predicted a shorter survival time for patients.^16^ To date, although one study has demonstrated the role of high expression of PPP1R14B in pancancer,^17^ no results have revealed the specific mechanism of PPP1R14B in UCEC, and its function in the development of UCEC remains unclear. This study was based on the analysis of online data without relevant experimental verification. Using the cervical cancer HeLa cell line and endometrial cancer HEC‐1‐A cell line, Xiang Nan et al.^18^ demonstrated that PPP1R14B knockdown could inhibit the activation of the Akt signalling pathway, thereby inhibiting cell proliferation and promoting cell death, but this study did not reveal a correlation of PPP1R14B with the clinical characteristics of tumours.

In this paper, we further verified the differential expression of the PPP1R14B gene in UCEC by mining the latest RNAseq data of UCEC in the TCGA database and verified its expression independently using the GEO database and Human Protein Atlas (HPA) database. In addition, 105 UCEC clinical samples were collected to further verify the expression of PPP1R14B in tumours and adjacent tissues, and the results of bioinformatics analysis were verified by experiments. Regarding the specific function of PPP1R14B in UCEC development and occurrence, protein–protein interaction (PPI) networks, gene essentiality (GO) terminology analysis, Kyoto Encyclopedia of Genes and Genomes (KEGG) pathway analysis, gene set enrichment analysis (GSEA), single‐sample gene set enrichment analysis (ssGSEA), and Kaplan–Meier survival analysis were used to predict PPP1R14B's role in UCEC patient prognosis. On the basis of a previous study,^17^, ^18^ the clinical characteristics of 105 clinical samples were used for correlation analysis, and we found that PPP1R14B expression was significantly correlated with FIGO grade and differentiation degree. Finally, we constructed a nomogram plot as a tool for clinicians to predict the prognosis of UCEC patients and help clinicians develop more suitable treatment plans for UCEC patients.

## 2 MATERIALS AND METHODS

### 2.1 Data sources and preprocessing

The differential RNAseq expression data of PPP1R14B in pancancer were obtained from UCSC XENA (https://xenabrowser.net/datapages/) in the TPM format of the TCGA and GTEx processed uniformly by the Toil process.^19^ The differential RNAseq expression data of PPP1R14B in unpaired and paired samples were in level 3 HTSeq‐FPKM format from the TCGA (https://portal.gdc.cancer.gov/) UCEC project. FPKM (Fragments Per Kilobase per Million) format RNAseq data were converted to TPM (transcripts per million reads) format and log2 transformed. All final analyses were performed using data in TPM format. The differential analysis data for PPP1R14B in dataset GSE17025^20^, ^21^ were downloaded from the GEO database using the GEOquery package (version 2.54.1).^22^ These data were obtained by removing probes corresponding to multiple molecules, and when probes corresponding to the same molecule were encountered, only the probe with the largest signal value was retained, and then the data were normalized again by the normalize Between Arrays function of the limma package (version 3.42.2).^23^ All statistical analyses and visualizations were performed using R (version 3.6.3).

### 2.2 Study population

The protocol of this retrospective study was approved by the Ethics Committee of the School of Nursing of Jilin University (Changchun, China) and was consistent with the principles of the Declaration of Helsinki. All enrolled patients were informed and agreed to participate in the present study and gave written informed consent. The paraffin‐embedded specimens of a total of 105 patients, all female, with UCEC who were diagnosed between 1 January 2019 and 31 May 2019 were collected from The Second Hospital of Jilin University (Changchun, China). The inclusion criteria were as follows: The first surgery was performed at the Second Hospital of Jilin University, and the pathological diagnosis was UCEC. The exclusion criteria were as follows: diagnosis of other malignant tumours; intrauterine device (IUD) and/or hormone therapy were used within 6 months before surgery. Ten specimens from UCEC patients of fresh‐frozen tumours and adjacent noncancerous tissue were collected between May 2021 and June 2022. Baseline patient characteristics and pathological data, including age, menopause status, differentiation degree, and FIGO stage, were extracted from the database of The Second Hospital of Jilin University.

### 2.3 Western blotting

Proteins were extracted from fresh‐frozen tissues followed by protein quantitation with a Coomassie Plus (Bradford) Assay Kit (Thermo Scientific, Cat#23236). Western blot analysis was conducted under standard procedures as previously described.^24^ The primary antibodies were PPP1R14B (Proteintech, Cat#18476‐1‐AP; Dilution, 1:500) and GAPDH (Proteintech, Cat#10494‐1‐AP; Dilution, 1:5000). The secondary antibody was HRP‐conjugated goat anti‐rabbit (Proteintech, Cat#SA00001‐2; Dilution, 1:20,000).

### 2.4 Immunohistochemistry

Immunohistochemical (IHC) staining assays were performed as previously described.^25^ Briefly, the paraffin‐embedded tissues were cut into 4 μm thick slides. After deparaffinization and rehydration, antigen retrieval was performed with each slide. Then, slides were blocked with 5% serum and incubated with primary antibodies against PPP1R14B (Servicebio, Cat#GB113534; Dilution, 1:2000) overnight at 4°C. The following procedure was performed: incubation with secondary antibodies, signal detection with DAB chromogen solution, counterstaining with haematoxylin, dehydration, and sealing with neutral gum. Finally, the slides were imaged using an Olympus optical microscope (BX51). The evaluation criteria were based on staining intensity and the proportion of positive tumour cells as previously described.^26^ For staining intensity: 0 (no colour), 1 (light yellow), 2 (yellow brown), and 3 (brown). For the proportion of positive tumour cells: 0 (<5%), 1 (5%–25%), 2 (26%–50%), 3 (51%–75%), and 4 (>75%). The scoring was evaluated by two independent pathologists who were blinded to the patients' UCEC status.

### 2.5 Differential expression analysis of PPP1R14B

The expression profiles of PPP1R14B across cancers were analysed for differences using the Mann–Whitney *U* test (Wilcoxon rank sum test). The Shapiro–Wilk normality test was used to test the normality of the PPP1R14B expression data in paired samples, unpaired samples, and GSE17025, and the independent samples *t* test was used to analyse the differences in the data in unpaired samples. The paired samples *t* test was used to analyse the differences in the data in paired samples, and the Mann–Whitney *U* test (Wilcoxon rank sum test) was used for analysis of variance of data in GSE17025. The results of all the above analyses were visualized using ggplot2 (version 3.3.3) and were considered statistically significant when *p* < 0.05.

### 2.6 Differential analysis of PPP1R14B protein expression levels in UCEC

Immunohistochemical staining images of PPP1R14B in UCEC and normal tissue sections were downloaded using the HPA database (https://www.proteinatlas.org/), where these sections were stained using the same antibodies and experimental methods.

### 2.7 Single‐gene differential analysis and correlation analysis of PPP1R14B

Single‐gene differential analysis of RNAseq data in level 3 HTSeq‐Counts format from the UCEC (endometrial cancer) project of TCGA was performed using the DESeq2 package (version 1.26.0).^27^ Single‐gene correlation analysis was performed on expression profile data in TPM format using the STAT package (version 3.6.3). The target molecule in the above analysis was PPP1R14B. Volcano plots were drawn using the results of single‐gene differential analysis, setting a threshold of |log2(FC)| > 1 and *p*.adj <0.05. These differentially expressed genes were entered into the STRING database,^28^ and protein–protein interaction (PPI) of differentially expressed genes was determined using Cytoscape software network analysis. Then, the HUB genes were identified using the MCODE plugin. Finally, using the results of single‐gene correlation analysis, the results were sorted by |Pearson value| in descending order, the genes whose correlations were in the top 50 were extracted, and the single‐gene coexpression heatmap of PPP1R14B was drawn using these genes and the HUB gene. Volcano plots and coexpression heatmaps were both generated by ggplot2 (version 3.3.3).

### 2.8 Functional enrichment analysis of PPP1R14B in UCEC

By using the clusterProfiler package (version 3.14.3), GO, KEGG, and GSEA functional enrichment analyses were performed on the results of single‐gene differential analysis.^29^ Gene ID conversion was performed using the org.Hs.eg.db package (version 3.10.0), and the *Z* score was calculated using the GOplot package (version 1.0.2),^30^ which scores the relevance of PPP1R14B to the enrichment pathway. The reference gene set used for the GSEA was c2.cgp.v7.2.symbols.gmt (Curated),^31^ and the results were significantly enriched if they met the conditions of false discovery rate (FDR) < 0.25 and *p*.adjust < 0.05. The visualization of all the above analysis results was performed using ggplot2 (version 3.3.3).

### 2.9 Immunoinfiltration analysis of PPP1R14B

The relative infiltration levels of 24 immune cells were analysed using the GSVA package (version 1.34.0).^32^ ssGSEA was performed for the algorithm of immune infiltration, and the chosen correlation analysis method was Spearman. Markers for 24 immune cells were obtained from an Immunity study.^33^ Afterwards, the samples were divided into low and high expression groups according to the expression of PPP1R14B, the enrichment scores of various immune cell infiltrates in the different subgroups were calculated, and the analysis was performed using the GSVA package (version 1.34.0). Finally, the correlation between immune cell infiltration and PPP1R14B expression was visualized by analysing immune cells with statistically significant relative infiltration (*p* < 0.001), and PPP1R14B gene expression data were used to draw chord diagrams. Statistical analysis and visualization were performed using the circlize package (version 0.4.12).^34^

### 2.10 Clinical correlation analysis, survival prognosis analysis, and construction of prognostic models

The survival data of UCEC patients were statistically analysed using the survival package (version 3.2‐10), and the results were visualized using the survminer package (version 0.4.9) to plot UCEC patients' overall survival (OS), disease‐specific survival (DSS), and progression‐free interval (PFI) of Kaplan–Meier survival curves. We then performed a subgroup analysis of Kaplan–Meier survival curves in UCEC patients for clinicopathological factors such as age, presence of diabetes, and menopause status. We then used these clinicopathological factors to calculate their correlation with PPP1R14B expression and visualized the calculated results using ggplot2 (version 3.3.3). ROC analysis of the data was performed using the pROC package (version 1.17.0.1) to determine the accuracy of PPP1R14B for prognostic prediction.

Finally, univariate and multivariate Cox regression analyses were performed again using the survival package (version 3.2‐10) for different clinicopathological factors and PPP1R14B expression, and the median was used to determine the critical value of PPP1R14B expression. The results were visualized by forest plots. Based on the results of the Cox regression analysis, a nomogram prognostic prediction model was constructed using the rms package (version 6.2‐0) and the survival package (version 3.2‐10), and a calibration plot was drawn to check the accuracy of the rainfall prediction. All prognostic data for the above survival analysis were obtained from a paper in Cell.^35^

### 2.11 Statistical analysis

Data are expressed as the mean ± standard deviation (mean ± SD). The difference in the expression of PPP1R14B in UCEC tumour tissues and adjacent tissues was analysed by Student's *t* test. One‐way analysis of variance (anova) was used for comparisons between multiple groups. The association between the expression of PPP1R14B and the clinical data of UCEC patients was analysed by Mann–Whitney *U* test analysis. The statistical graph was completed using GraphPad Prism 8, and *p* < 0.05 was regarded as statistically significant.

## 3 RESULTS

### 3.1 Differential expression of PPP1R14B in pancancer and UCEC

**FIGURE 1:**
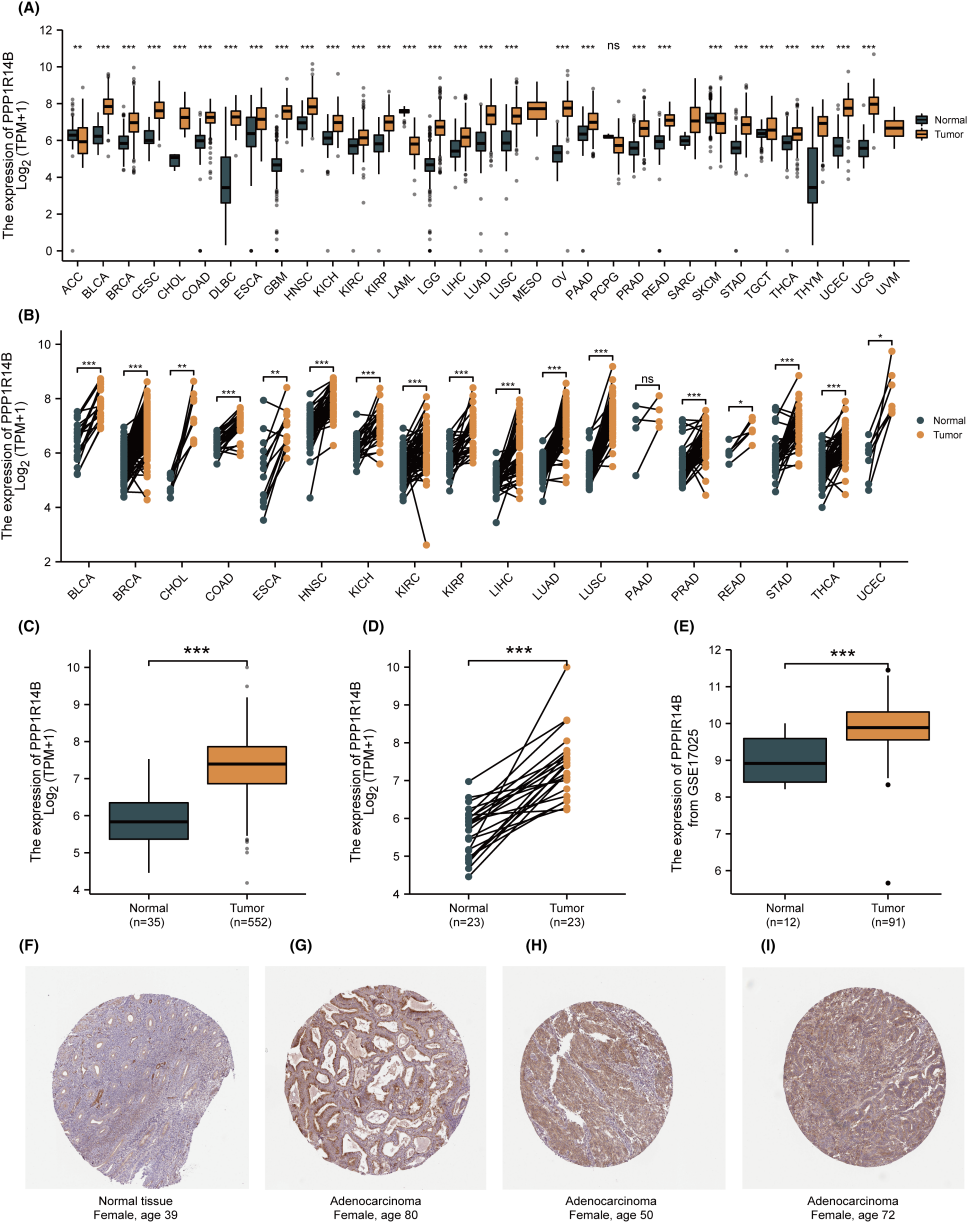
Differential expression of PPP1R14B in pancancer and uterine corpus endometrial carcinoma (UCEC). (A) Results of differential analysis of PPP1R14B expression in 33 tumours based on the data in the TCGA database. (B) Differential analysis of PPP1R14B expression based on paired pancancer tumour data from the TCGA database. (C) Results of differential analysis of PPP1R14B expression in unpaired samples. (D) Results of differential analysis of PPP1R14B expression in paired samples. (E) Results of differential analysis of PPP1R14B expression based on the data in dataset GSE17025. (F–I) Immunohistochemical images of protein expression in normal tissue (F) versus UCEC (G–I) in the Human Protein Atlas (HPA) data.

The results of differential expression analysis of PPP1R14B across carcinomas are shown in Figure 1A. In adrenocortical carcinoma (ACC, *T* = 77, *N* = 128), acute myeloid leukaemia (LAML, *T* = 173, *N* = 70), and skin cutaneous melanoma (SKCM, *T* = 469, *N* = 813), the expression of PPP1R14B was lower than that in normal tissues, and the differences were statistically significant (*p* < 0.05). In bladder urothelial carcinoma (BLCA, *T* = 407, *N* = 28), breast invasive carcinoma (BRCA, *T* = 1099, *N* = 292), cervical squamous cell carcinoma, and endocervical adenocarcinoma (CESC, *T* = 306, *N* = 13), the cholangiocarcinoma (CHOL, *T* = 36, *N* = 9), colon adenocarcinoma (COAD, *T* = 290, *N* = 349), Neoplasm Diffuse Large B‐cell Lymphoma (DLBC, *T* = 47, *N* = 444), oesophageal carcinoma (ESCA, *T* = 182, *N* = 666), glioblastoma multiforme (GBM, *T* = 166, *N* = 1157), head and neck squamous cell carcinoma (HNSC, *T* = 520, *N* = 44), kidney chromophobe (KICH, *T* = 66, *N* = 53), kidney renal clear cell carcinoma (KIRC, *T* = 531, *N* = 100), kidney renal papillary cell carcinoma (KIRP, *T* = 289, *N* = 60), brain lower grade glioma (LGG, *T* = 523, *N* = 1152), liver hepatocellular carcinoma (LIHC, *T* = 371, *N* = 160), lung adenocarcinoma (LUAD, *T* = 515, *N* = 347), lung squamous cell carcinoma (LUSC, *T* = 498, *N* = 338), ovarian serous cystadenocarcinoma (OV, *T* = 427, *N* = 88), pancreatic adenocarcinoma (PAAD, *T* = 179, *N* = 171), prostate adenocarcinoma (PRAD, *T* = 496, *N* = 152), rectum adenocarcinoma (READ, *T* = 93, *N* = 318), stomach adenocarcinoma (STAD, *T* = 414, *N* = 210), testicular germ cell tumours (TGCT, *T* = 154, *N* = 165), thyroid carcinoma (THCA, *T* = 512, *N* = 338), thymoma (THYM, *T* = 119, *N* = 446), uterine corpus endometrial carcinoma (UCEC, *T* = 181, *N* = 101), and uterine carcinosarcoma (UCS, *T* = 57, *N* = 78), the expression of PPP1R14B was higher than that of normal tissues, and the difference was statistically significant (*p* < 0.05). As shown in Figure 1B, in pancancer pairs, the expression of PPP1R14B in BLCA (*T* = 19, *N* = 19), BRCA (*T* = 112, *N* = 112), CHOL (*T* = 9, *N* = 9), COAD (*T* = 26, *N* = 26), ESCA (*T* = 13, *N* = 13), HNSC (*T* = 43, *N* = 43), KICH (*T* = 25, *N* = 25), KIRC (*T* = 72, *N* = 72), KIRP (*T* = 32, *N* = 32), LIHC (*T* = 50, *N* = 50), LUAD (*T* = 58, *N* = 58), LUSC (*T* = 50, *N* = 50), PAAD (*T* = 4, *N* = 4), PRAD (*T* = 52, *N* = 52), READ (*T* = 6, *N* = 6), STAD (*T* = 33, *N* = 33), THCA (*T* = 59, *N* = 59), and UCEC (*T* = 7, *N* = 7) was higher than that in the adjacent tissue, and the difference was statistically significant (*p* < 0.05). In both paired and unpaired samples of UCEC, the expression of PPP1R14B was significantly different compared with that in normal samples, and the results are shown in Figure 1C,D. We then used the GSE17025 dataset from the GEO database to verify the results in the TCGA database, and the results are shown in Figure 1E; the results were still significantly different. Finally, we analysed the difference in the protein expression levels of PPP1R14B in UCEC tissues and normal tissues using the HPA online database, and their immunohistochemical (IHC) images are shown in Figure 1F–I. IHC images of normal endometrium are presented in Figure 1F, while IHC images are presented in Figure 1G–I of endometrial adenocarcinoma tissue from an 80‐year‐old woman, a 50‐year‐old woman, and a 72‐year‐old woman, respectively. The expression of PPP1R14B was not detected in normal tissues, but high expression of PPP1R14B was detected in tumour tissues.

### 3.2 Single‐gene differential analysis and correlation analysis of PPP1R14B

**FIGURE 2:**
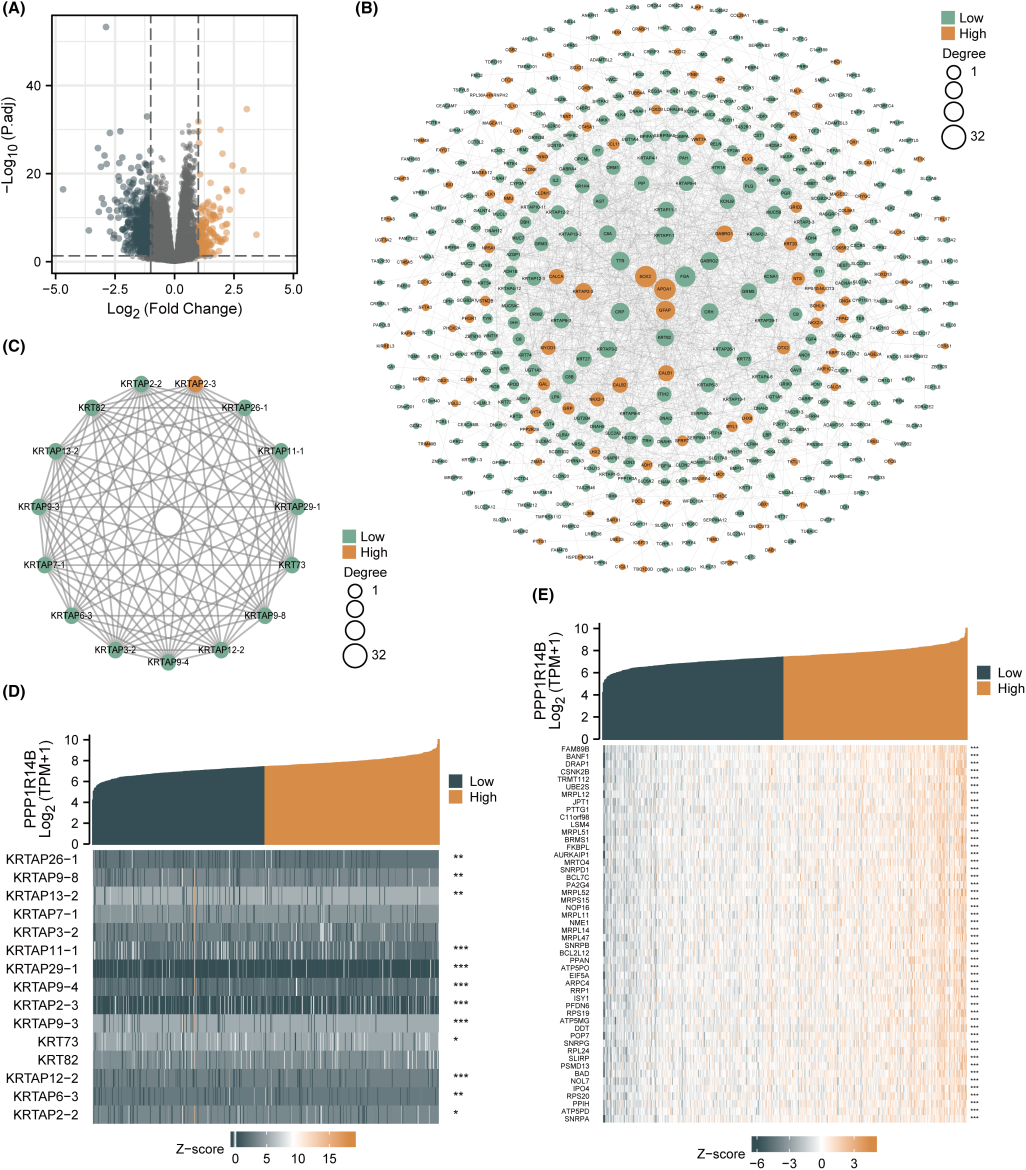
Single‐gene differential analysis and correlation analysis of PPP1R14B. (A) Volcano plot of single gene differential analysis of PPP1R14B. (B) Protein–protein interaction network (PPI) of differentially expressed genes in single gene differential analysis. (C) Protein–protein interaction network map (PPI) of the HUB gene. (D) Single gene coexpression heatmap of the HUB gene and PPP1R14B. (E) Single gene coexpression heatmap of the top 50 most strongly correlated genes with PPP1R14B in single gene correlation analysis.

Single gene differential analysis of PPP1R14B was performed in UCEC, and the results are shown in Figure 2A. A total of 687 genes satisfied the threshold of |log2(FC)| > 1 and *p*.adj < 0.05, and under this threshold, the number of genes with high expression (positive log2FC) was 161, and the number of genes with low expression (negative log2FC) was 526. We then constructed a PPI network using these 687 differentially expressed genes, and the results are shown in Figure 2B. The closer the genes are to the centre of the interaction network graph, the more connections they have with other genes. Then, using the MCODE plugin, we identified 15 HUB genes, namely, KRTAP26‐1, KRTAP9‐8, KRTAP13‐2, KRTAP7‐1, KRTAP3‐2, KRTAP11‐1, KRTAP29‐1, KRTAP9‐4, KRTAP2‐3, KRTAP9‐3, KRTAP KRT73, KRT82, KRTAP12‐2, KRTAP6‐3, and KRTAP2‐2. The PPI network of HUB genes is shown in Figure 2C. These hub genes were all keratin‐associated proteins or keratins, and based on these genes, we drew their gene coexpression heatmap with PPP1R14B, as shown in Figure 2D. Finally, we performed single gene correlation analysis of PPP14R14B and selected the top 50 most strongly correlated genes to draw a correlation heatmap with PPP1R14B, and the results are shown in Figure 2E.

### 3.3 Functional enrichment analysis of PPP1R14B in UCEC

**FIGURE 3:**
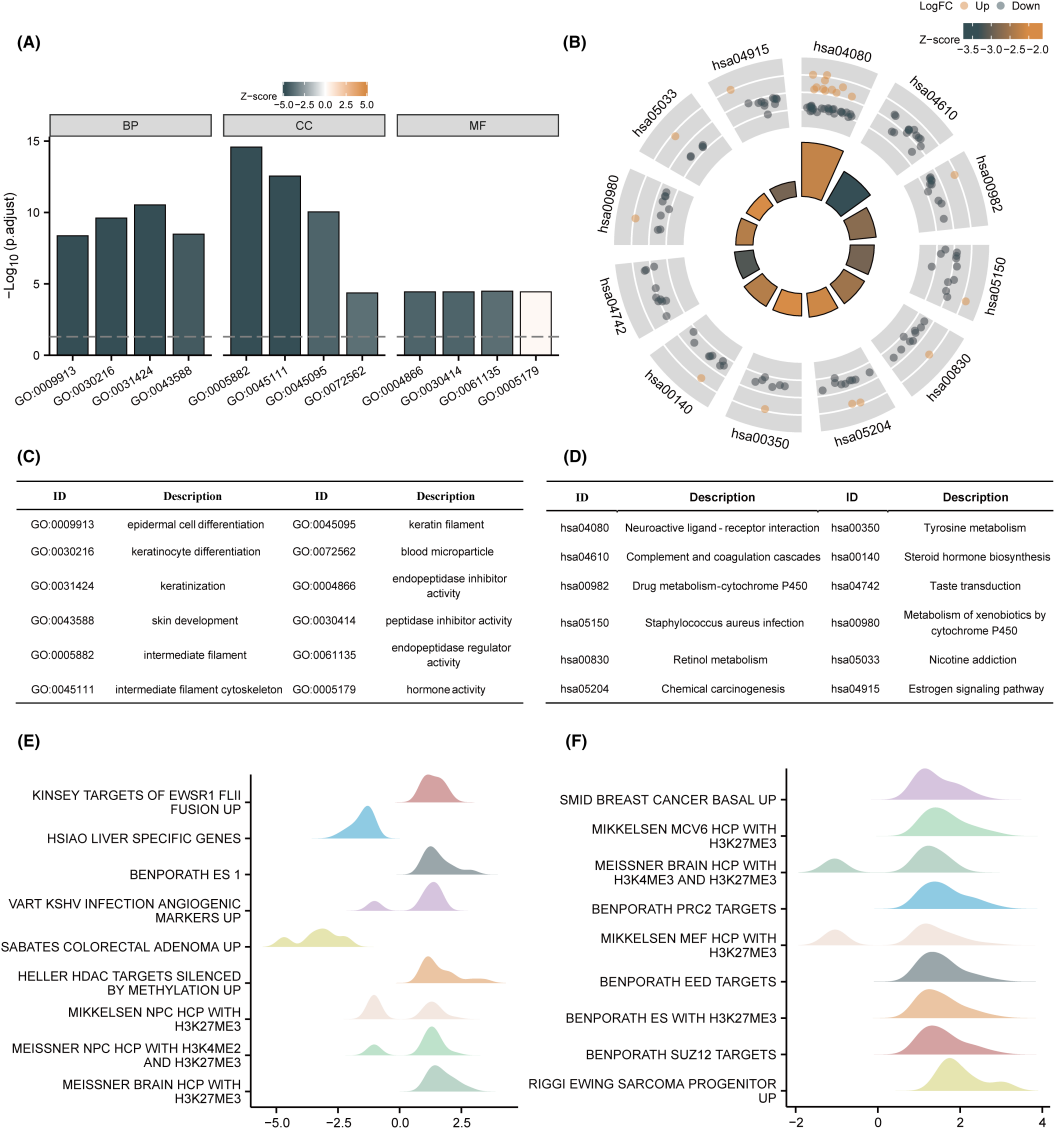
Functional enrichment analysis of PPP1R14B in uterine corpus endometrial carcinoma. (A) Results of GO analysis. (B) Results of KEGG analysis. (C, D) GO and KEGG analysis category names corresponding to GO and KEGG identifiers. (E, F) The results of GSEA. When the horizontal coordinate is positive, it suggests that the expression of PPP1R14 is positively correlated with the pathway, while the opposite is true when the horizontal coordinate is negative.

GO, KEGG, and GSEA enrichment analyses were performed using the results of single‐gene differential analysis, and the results are shown in Figure 3. Figure 3A,C and Table 1 show the results of GO analysis, which revealed that PPP1R14B is functionally related to epidermal cell differentiation, endopeptidase activity regulation, blood microparticles, and hormone activity. Figure 3B,D and Table 1 show the results of KEGG analysis, which revealed that PPP1R14B was associated with neuroactive ligand–receptor interaction, retinol metabolism, chemical carcinogenesis, tyrosine metabolism, steroid hormone biosynthesis, oestrogen signalling pathway, etc. The *Z* score reflects the correlation of PPP1R14B with these pathways to some extent. A negative *Z* score indicates a negative correlation, and a positive *Z* score indicates a positive correlation. Figure 3E,F show the enrichment and grading results of GSEA, which suggested that there was significant enrichment in kinsey targets of ewsr1 flii fusion up, hsiao liver specific genes, benporath es 1, vart kshv infection angiogenic markers up, sabates colorectal adenoma up, heller hdac targets silenced by methylation up, and other genes related to tumorigenesis, invasion, and angiogenesis, suggesting that PPP1R14B was indeed closely related to cancer.

**TABLE 1:** GO and KEGG analysis.

Ontology	ID	Description	Gene Ratio	Bg ratio	*p* Value	*p*.Adjust	*q* Value
CC	GO:0005882	Intermediate filament	37/598	214/19,717	6.78e‐18	2.65e‐15	2.35e‐15
CC	GO:0045111	Intermediate filament cytoskeleton	37/598	251/19,717	1.45e‐15	2.83e‐13	2.52e‐13
BP	GO:0009913	Epidermal cell differentiation	39/565	358/18,670	4.59e‐12	4.28e‐09	4.04e‐09
BP	GO:0030216	Keratinocyte differentiation	38/565	305/18,670	1.33e‐13	2.48e‐10	2.34e‐10
BP	GO:0031424	Keratinization	34/565	224/18,670	7.83e‐15	2.92e‐11	2.76e‐11
BP	GO:0043588	Skin development	43/565	419/18,670	2.68e‐12	3.32e‐09	3.14e‐09
CC	GO:0045095	Keratin filament	21/598	95/19,717	6.99e‐13	9.09e‐11	8.07e‐11
MF	GO:0004866	Endopeptidase inhibitor activity	21/548	175/17,697	1.17e‐07	3.59e‐05	3.04e‐05
MF	GO:0030414	Peptidase inhibitor activity	21/548	182/17,697	2.30e‐07	3.59e‐05	3.04e‐05
MF	GO:0061135	Endopeptidase regulator activity	22/548	182/17,697	5.14e‐08	3.22e‐05	2.72e‐05
CC	GO:0072562	Blood microparticle	18/598	147/19,717	5.23e‐07	4.33e‐05	3.85e‐05
MF	GO:0005179	Hormone activity	17/548	122/17,697	2.19e‐07	3.59e‐05	3.04e‐05
KEGG	hsa04080	Neuroactive ligand‐receptor interaction	35/266	341/8076	1.45e‐09	3.57e‐07	3.38e‐07
KEGG	hsa04610	Complement and coagulation cascades	14/266	85/8076	5.88e‐07	7.23e‐05	6.84e‐05
KEGG	hsa00982	Drug metabolism ‐ cytochrome P450	11/266	71/8076	1.78e‐05	0.001	0.001
KEGG	hsa05150	Staphylococcus aureus infection	12/266	96/8076	6.75e‐05	0.003	0.003
KEGG	hsa00830	Retinol metabolism	10/266	68/8076	6.83e‐05	0.003	0.003
KEGG	hsa05204	Chemical carcinogenesis	11/266	82/8076	7.07e‐05	0.003	0.003
KEGG	hsa00350	Tyrosine metabolism	7/266	36/8076	1.43e‐04	0.005	0.004
KEGG	hsa00140	Steroid hormone biosynthesis	9/266	61/8076	1.54e‐04	0.005	0.004
KEGG	hsa04742	Taste transduction	10/266	86/8076	4.94e‐04	0.014	0.013
KEGG	hsa00980	Metabolism of xenobiotics by cytochrome P450	9/266	77/8076	9.05e‐04	0.022	0.021
KEGG	hsa05033	Nicotine addiction	6/266	40/8076	0.002	0.040	0.037
KEGG	hsa04915	Oestrogen signalling pathway	12/266	138/8076	0.002	0.040	0.037

Abbreviations: BP, biological process; CC, cellular component; MF, molecular function.

### 3.4 Immunoinfiltration analysis of PPP1R14B

**FIGURE 4:**
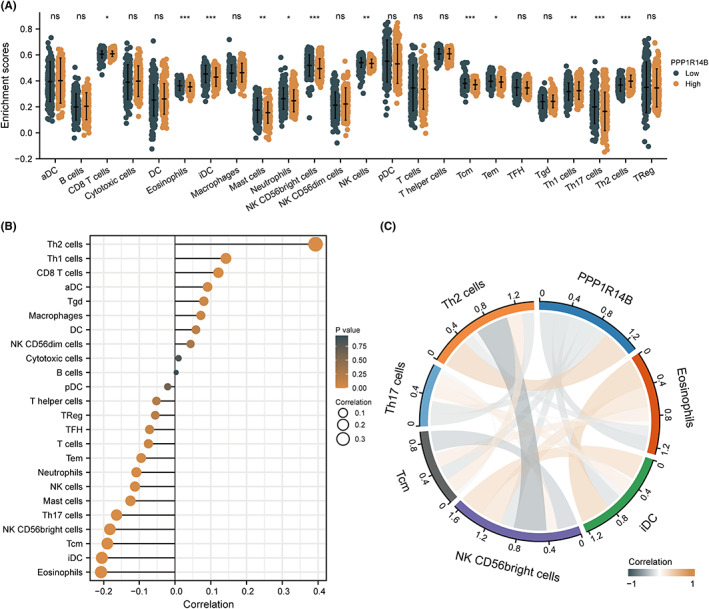
Immune infiltration analysis of PPP1R14B. (A) Grouped comparison of the infiltration levels of 24 immune cells in the low and high expression groups of PPP1R14B. (B) Results of ssGSEA of the correlation between the expression of PPP1R14B and 24 immune cells. (C) Chord plot of the correlation between PPP1R14B and infiltration levels of eosinophils, iDCs, NK CD56bright cells, Tcm, Th17 cells, and Th2 cells, with darker colour representing stronger correlation, brown representing positive correlation and dark blue representing negative correlation.

To determine the effect of PPP1R14B expression on the tumour microenvironment, immune infiltration analysis was performed using the ssGSEA method. The correlation between immune cell enrichment and PPP1R14B expression levels in UCEC tissues was calculated using Spearman correlation analysis. The results are shown in Figure 4B. The expression of PPP1R14B was positively correlated with the level of infiltration of four immune cells, namely, aDCs, CD8 T cells, Th1 cells, and Th2 cells. The expression of PPP1R14B was negatively correlated with the level of infiltration of nine immune cells, namely, eosinophils, iDCs, mast cells, neutrophils NK CD56bright cells, NK cells, Tcm, Tem, and Th17 cells. Next, we divided the expression profile data into high and low expression groups according to the expression level of PPP1R14B to identify the changes in the level of immune cell infiltration in the different groups. The results, which are shown in Figure 4A, indicate that in CD8 T cells, Th1 cells, and Th2 cells, the level of infiltration in the low expression group was significantly lower than that in the high expression group. In eosinophils, iDCs, mast cells, neutrophils, NK CD56bright cells, NK cells, Tcm cells, Tems, and Th17 cells, the infiltration level of the low expression group was significantly higher than that of the high expression group, and this result is consistent with the results shown in Figure 4B. Finally, we used the expression values of PPP1R14B with the enrichment scores of the six immune cells with significant correlation to produce chord plots to visualize the correlation between them, and the results are shown in Figure 4C.

### 3.5 Prognostic analysis of survival of PPP1R14B expression

**FIGURE 5:**
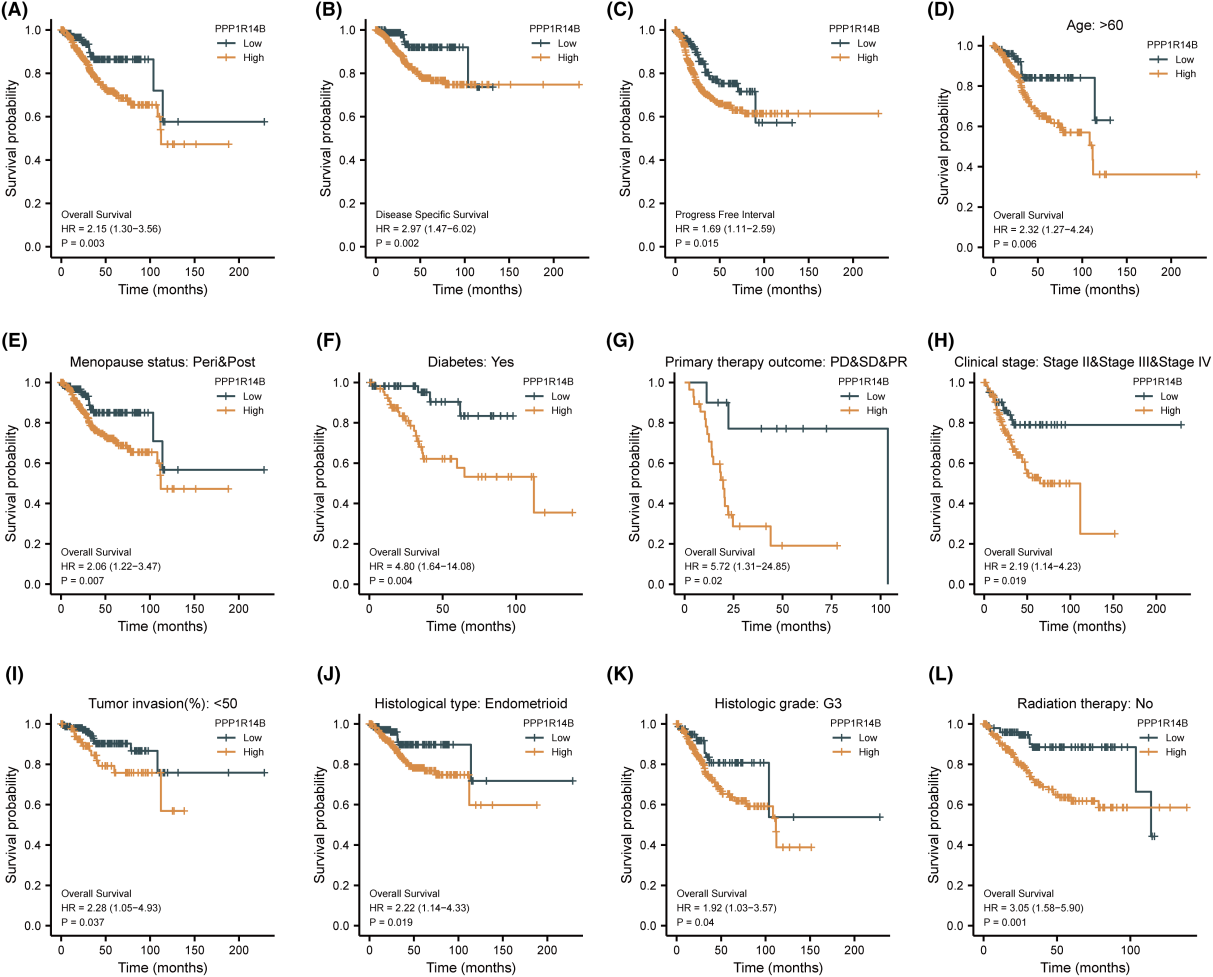
Prognostic analysis of survival and PPP1R14B expression. (A–C) Kaplan–Meier survival curves of the relationship between PPP1R14B expression and overall survival (OS), disease‐specific survival (DSS), and progression‐free interval (PFI). (D–L) Kaplan–Meier survival curves of the relationship between PPP1R14B expression and OS in different patient subgroups with distinct clinicopathological factors.

First, we divided the expression profile data into high and low expression groups according to the expression of PPP1R14B and analysed the overall survival (OS), disease‐specific survival (DSS), and progression‐free interval (PFI) of UCEC patients in the different groups. The results showed that the groups with high expression of PPP1R14B all exhibited shorter survival times, as shown in Figure 5A–C. We then grouped the clinicopathological factors to analyse whether the OS of UCEC patients would be significantly different in the different subgroups. We found that among patients older than 60 years, among patients with menopause status in peri and post, patients with diabetes, patients with primary therapy outcome of PD, SD, or PR, patients with clinical stage II, stage III, or stage IV, patients with less than 50% tumour invasion, patients with histologic type endometrioid, patients with histologic grade G3 level, and patients who did not receive radiation therapy, the group with high expression of PPP1R14 showed shorter survival time (Figure 5D–L). These results suggested that high expression of PPP1R14B was associated with poor prognosis and was closely related to tumour development. Furthermore, these findings indicated that high expression of PPP1R14B is a risk factor for patients.

### 3.6 Clinical correlation analysis of PPP1R14B expression

**FIGURE 6:**
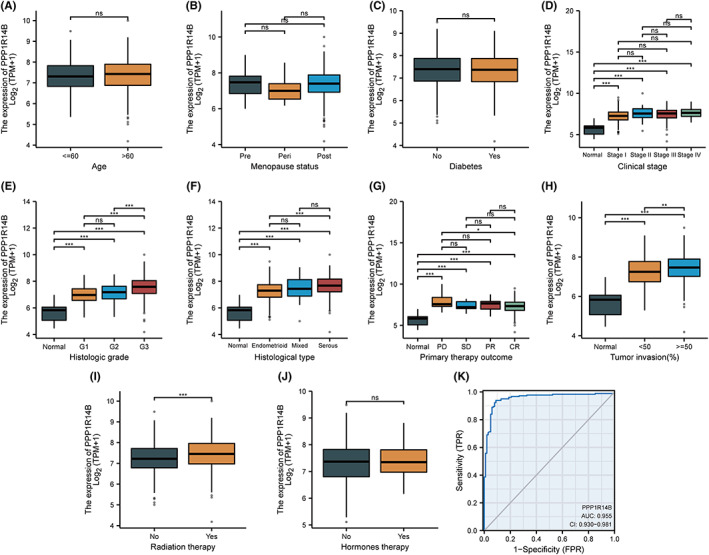
Clinical correlation analysis of PPP1R14B expression. (A–J) Expression levels of PPP1R14B in different groups of patients with distinct clinicopathological factors. (K) ROC curve, the area under the ROC curve is between 0.5 and 1. The closer the AUC is to 1, the better the diagnosis is. The AUC is between 0.5 and 0.7 with low accuracy, the AUC is between 0.7 and 0.9 with some accuracy, and the AUC is above 0.9 with high accuracy.

To further verify that high expression of PPP1R14B is associated with poor prognosis and is closely related to tumour development, we compared the expression levels of PPP1R14B in patients from different groups with distinct clinicopathological factors, and the results are shown in Figure 6A–J. A clinical baseline information table was constructed based on the expression level of PPP1R14B, as shown in Table 2. The expression levels of PPP1R14B in patients with different clinical stages or different histological types, etc., were significantly different from those in control patients, which suggested that the expression of PPP1R14B may be associated with tumorigenesis and can be used as a marker for tumour diagnosis. Then, we found a significant difference in PPP1R14B expression between patients with endometrioid and serous histological types of tumours, suggesting that PPP1R14B is indeed associated with poor prognosis. The expression of PPP1R14B also differed between patients with primary therapy outcomes of PD and CR, suggesting that its expression may also be associated with disease progression. The expression levels of PPP1R14B also differed among patients with different levels of tumour invasion, suggesting that the level of tumour invasion may also be correlated with the expression of PPP1R14. Finally, we constructed a ROC curve to verify the accuracy of PPP1R14B expression in predicting prognosis, and the results are shown in Figure 6K. The predictive ability of the variable PPP1R14B was somewhat accurate in predicting the prognostic outcome of tumour patients versus normal patients (AUC = 0.955, CI = 0.930–0.981).

**TABLE 2:** Baseline data sheet.

Characteristic	Low expression of PPP1R14B	High expression of PPP1R14B	*p*
*n*	276	276	
Age, *n* (%)^a^			
≤60	107 (19.5%)	99 (18%)	0.605
>60	169 (30.8%)	174 (31.7%)	
Menopause status, *n* (%)^a^			
Pre	14 (2.8%)	21 (4.2%)	0.117
Peri	12 (2.4%)	5 (1%)	
Post	226 (44.7%)	228 (45.1%)	
Diabetes, *n* (%)^a^			
No	161 (35.7%)	167 (37%)	0.448
Yes	66 (14.6%)	57 (12.6%)	
Clinical stage, *n* (%)			
Stage I	195 (35.3%)	147 (26.6%)	<0.001
Stage II	21 (3.8%)	30 (5.4%)	
Stage III	52 (9.4%)	78 (14.1%)	
Stage IV	8 (1.4%)	21 (3.8%)	
Histologic grade, *n* (%)^a^			
G1	72 (13.3%)	26 (4.8%)	<0.001
G2	77 (14.2%)	43 (7.9%)	
G3	124 (22.9%)	199 (36.8%)	
Histological type, *n* (%)			
Endometrioid	224 (40.6%)	186 (33.7%)	<0.001
Mixed	11 (2%)	13 (2.4%)	
Serous	41 (7.4%)	77 (13.9%)	
Primary therapy outcome, *n* (%)^a^			
PD	7 (1.5%)	13 (2.7%)	0.206
SD	4 (0.8%)	2 (0.4%)	
PR	4 (0.8%)	8 (1.7%)	
CR	232 (48.3%)	210 (43.8%)	
Tumour invasion (%), *n* (%)^a^			
<50	150 (31.6%)	109 (23%)	0.005
≥50	96 (20.3%)	119 (25.1%)	
Radiation therapy, *n* (%)^a^			
No	160 (30.4%)	119 (22.6%)	0.009
Yes	113 (21.4%)	135 (25.6%)	
Hormones therapy, *n* (%)^a^			
No	154 (44.8%)	143 (41.6%)	0.989
Yes	25 (7.3%)	22 (6.4%)	
Age, median (IQR)	63 (57, 71)	64 (57, 72)	0.553

Abbreviations: CR, complete response; PD, progressive disease; PR, partial response; SD, stable disease.

^a^ Data incomplete as some record data were lost in the online database.

### 3.7 Construction and verification of the nomogram based on PPP1R14B

**FIGURE 7:**
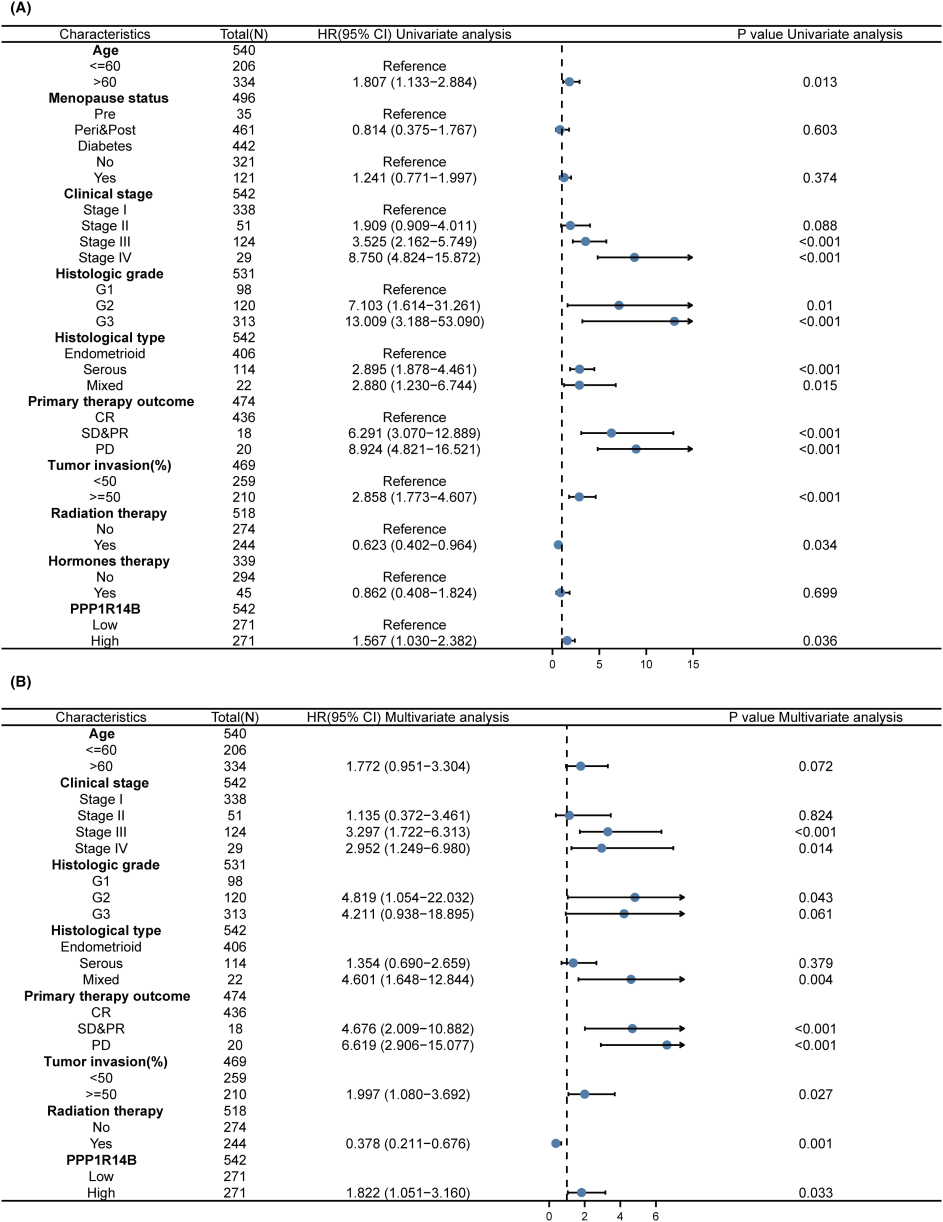
Results of Cox regression analysis of different clinicopathological factors and expression values of PPP1R14B. (A) Results of single‐factor analysis. (B) Results of multifactor analysis.

**FIGURE 8:**
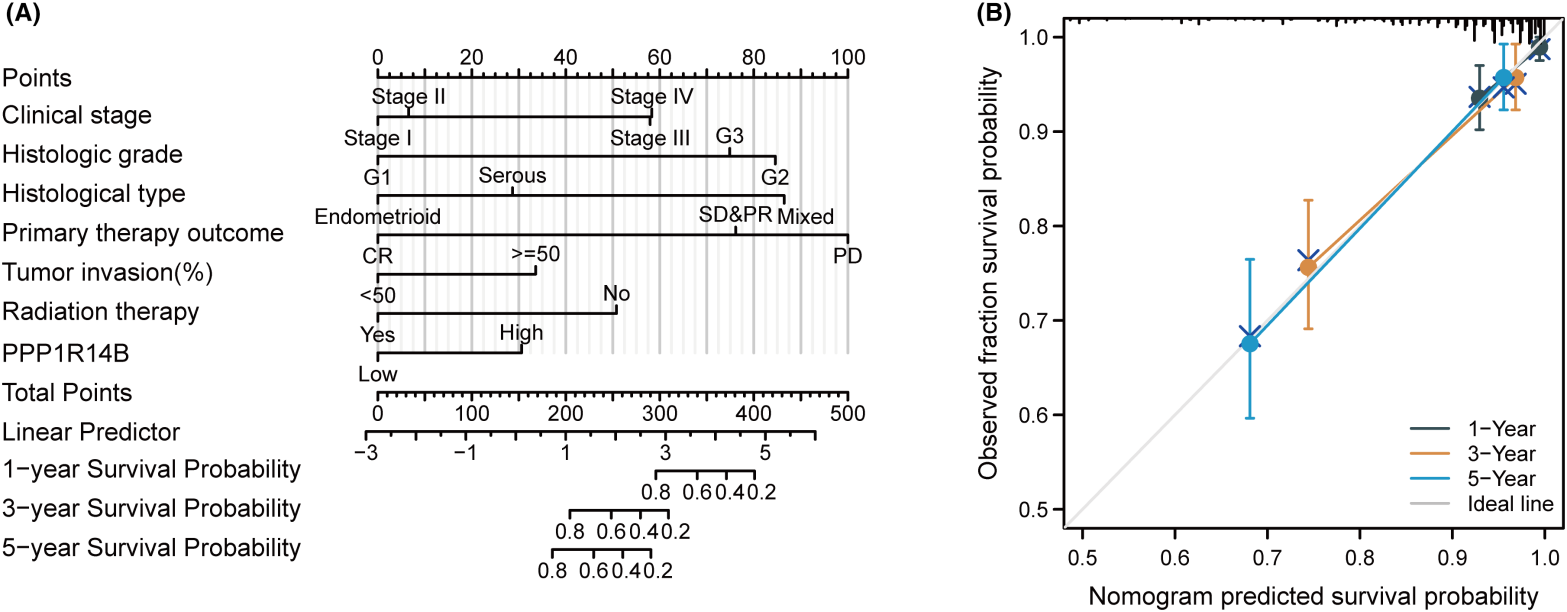
Construction and verification of the nomogram based on PPP1R14B expression. (A) nomogram model. (B) Calibration graph.

To construct an easy‐to‐use nomogram graph as a tool for clinicians to judge prognosis, we first performed univariate and multivariate COX regression analyses using different clinicopathological factors with PPP1R14B expression values to find independent prognostic factors for UCEC patients. The results are shown in Figure 7 and reveal that high expression of PPP1R14B is an independent prognostic risk factor for UCEC patients. The nomogram we constructed is shown in Figure 8A, and the factors used for prediction included clinical stage, histologic grade, histological type, primary therapy outcome, tumour invasion, and radiation therapy with the expression of PPP1R14B. To verify the accuracy of this prediction tool to predict prognosis, we constructed a calibration plot, as shown in Figure 8B, with a concordance index (C index) of 0.83, indicating a moderate accuracy of predictive ability, and the calibration plot was very close to the diagonal, which indicated good calibration performance.

### 3.8 Evaluation of the expression of PPP1R14B in clinical samples of UCEC

**FIGURE 9:**
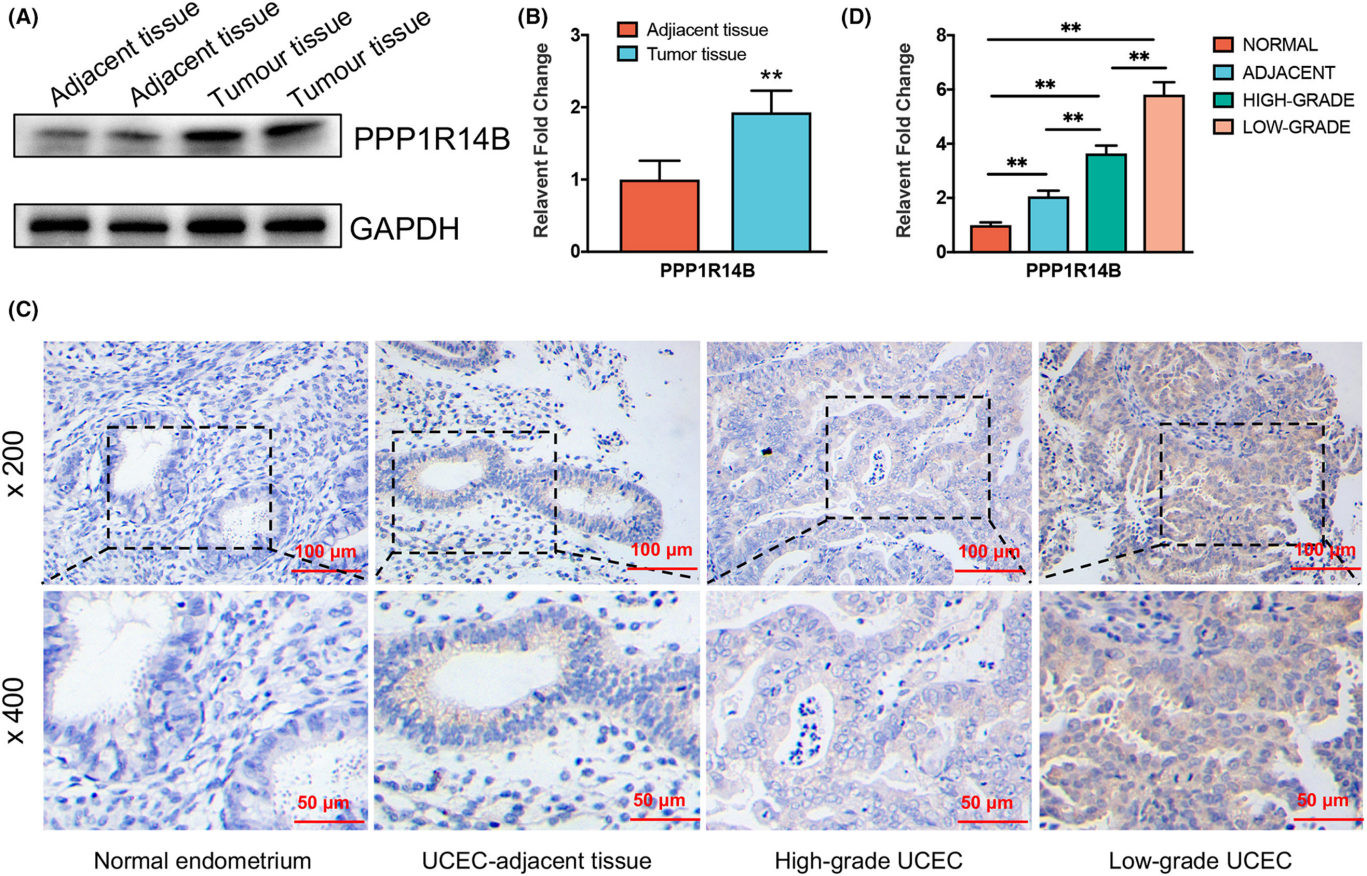
Evaluation of the expression of PPP1R14B in clinical uterine corpus endometrial carcinoma (UCEC) samples. (A, C) The expression of PPP1R14B and GAPDH in UCEC and UCEC‐adjacent tissues detected by Western blot and the statistical results of Western blot. (B) Immunohistochemical staining of PPP1R14B in normal endometrium, UCEC‐adjacent tissue, high‐grade UCEC, and low‐grade UCEC. (D) The statistical results of immunohistochemical staining. (*p < 0.05, **p < 0.01).

Next, we evaluated the expression of PPP1R14B in 10 UCEC tumour tissues and 10 adjacent tissues by Western blot analysis. As shown in Figure 9 A and B, the expression of PPP1R14B was higher in UCEC tumour tissues than in adjacent tissues. Additionally, the results of IHC staining confirmed the upregulated expression of PPP1R14B in low‐grade UCEC (Figure 9B–D). All clinical data, including age, menopause status, pathological grade, and FIGO stage, are summarized in Table 3. After Mann–Whitney *U* test analysis, pathological grade and FIGO stage were determined to be related to the level of PPP1R14B expression. UCEC patients with low pathological grade (*p* < 0.05) or high FIGO stage (*p* < 0.05) showed higher expression of PPP1R14B.

**TABLE 3:** Association between the expression of PPP1R14B and clinical features of samples with uterine corpus endometrial carcinoma.

Characteristic	Low expression of PPP1R14B	High expression of PPP1R14B	*p*
*n*	62	43	
Age, *n* (%)			
≤55	34 (32.4%)	20 (19.0%)	0.403
>55	28 (26.7%)	23 (21.9%)	
Menopause status, *n* (%)			
Yes	42 (40%)	30 (28.6%)	0.827
No	20 (19%)	13 (12.4%)	
Pathological grade, *n* (%)			
Low level	23 (21.9%)	25 (23.8%)	0.034
High level	39 (37.1%)	18 (17.2%)	
FIGO stage, *n* (%)			
I	53 (50.5%)	30 (28.6%)	0.041
II	4 (3.8%)	3 (2.8%)	
III	5 (4.8%)	10 (9.5%)	

Abbreviation: FIGO, Federation International of Gynaecology and Obstetric.

## 4 DISCUSSION

Uterine corpus endometrial carcinoma is one of the most common gynaecological malignancies. In recent years, the incidence and related mortality of UCEC has been increasing year by year, and a trend toward younger age‐of‐onset has also emerged.^36^ Epidemiological results showed that 6.5% of patients with UCEC were younger than 45 years of age, and nearly 70% of them were diagnosed before first pregnancy.^37^ How to develop personalized treatment programs to preserve fertility, reduce mortality, and improve life quality will be the focus and theme of more research in the future.

Therefore, further exploration of more molecular biomarkers is of great importance to detect the occurrence and prognosis of UCEC and develop more reasonable treatment plans. In this study, UCEC expression profile data from the TCGA and GEO databases were used to screen differentially expressed genes between UCEC and normal tissues. PPP1R14B expression in UCEC was higher than that in normal tissues, suggesting that PPP1R14B may be involved in the occurrence and development of tumours. Similar to this study, Mingxia Deng et al.^17^ found that PPP1R14B in pancancer was a type of diagnostic molecular marker associated with immune infiltration.

To further predict the molecular mechanism by which PPP1R14B promotes the occurrence and development of endometrial cancer, single‐gene differential analysis and correlation analysis were performed, and a PPI protein interaction network was constructed. Fifteen HUB genes most related to PPP1R14B expression and 50 genes closely related to PPP1R14B expression were found to predict the function of the PPP1R14B gene. Among them, keratin‐related proteins and keratin‐PPP1R14B functions were particularly closely related to PPP1R14B expression. Keratin‐associated proteins (KRTAPs) comprise a large multigene family of proteins thought to be responsible for the bundling of keratin intermediate filaments.^38^ Keratin (KRT) is a cytoskeletal protein of epithelial cells^39^ that is involved in the regulation of apoptosis tolerance, growth, and migration of tumour cells and is closely related to tumorigenesis. Some keratin is increased in the serum of tumour patients or highly expressed in tumour tissues, which is widely used in the diagnosis of tumours clinically. Keratin expression is negatively correlated with the prognosis of tumour patients and can be used as a prognostic marker.^40^, ^41^, ^42^, ^43^ In addition, keratin 82 (KRT82) mutations have been shown to be prevalent in gastric, colorectal, and endometrial cancers.^44^, ^45^ Keratin‐associated protein 6–3 (KRTAP6‐3) mutations have been found in patients with aggressive brain tumours.^46^ Therefore, we hypothesized that PPP1R14B may play a role in promoting endometrial cancer by promoting the function of keratin‐related proteins and keratin. In addition, among the genes closely associated with PPP1R14B expression, UBE2S, JPT1, and PTTG1 were associated with abnormal expression of endometrial cancer desiccations, proliferation, migration, methylation, and prediction of response to metformin therapy.^47^, ^48^, ^49^, ^50^ Our study suggested that the high expression of PPP1R14B may be related to gene mutation and tumour proliferation, invasion, and metastasis in UCEC patients.

To further clarify the possible molecular mechanism by which PPP1R14B promotes tumour development, GO analysis, KEGG analysis, and GSEA were performed. The results showed that PPP1R14B was negatively associated with intermediate filament‐related pathways, and intermediate filaments could inhibit UCEC metastasis and invasion.^51^, ^52^ In addition, hormone activity and oestrogen signalling pathways were found to be enriched. Given that endometrial cancer formation is associated with dysplasia caused by oestrogen overstimulation, this result suggests that involvement in this process may also be one of the mechanisms by which PPP1R14B promotes endometrial cancer development. In addition, the enrichment of retinol metabolism and tyrosine metabolism has been observed, and retinol and its derivatives can effectively delay or prevent precancerous lesions and induce tumour cell differentiation and apoptosis.^53^ Abnormal tyrosine metabolism also plays a very important role in the occurrence and development of tumours.^54^ GSEA results showed that PPP1R14B was closely related to H3K27me3, PRC2, and other epigenetic genes, which are closely related to the stem cell characteristics of tumour cells. In many poorly differentiated tumours, the stem cell phenotype is often very obvious. These results suggest that PPP1R14B may contribute to UCEC tumour cells showing stem cell characteristics, which are closely related to tumour differentiation and proliferation.^55^, ^56^, ^57^ Further experimental studies are needed to confirm the underlying mechanism of high expression of these pathways in PPP1R14B, leading to poor prognosis in UCEC patients.

The correlation between PPP1R14B expression and 24 types of immune cells in UCEC patients was also analysed. The results showed that PPP1R14B expression was negatively correlated with eosinophils, iDCs, mast cells, neutrophils, and other immune cells. Immune cell infiltration can improve the poor prognosis of patients, and low concentrations of infiltrating immune cells can lead to immune escape of cancer cells, resulting in poor prognosis.^58^, ^59^ In addition, the results of immune infiltration showed that the degree of Th2 cell infiltration was significantly positively correlated with the expression of PPP1R14B. The transition from Th1/Th2 balance to Th2 dominance is a crucial factor in tumour progression, and Th2 cells are not conducive to the antitumour effect of cellular immunity. Restoring the balance between Th1 and Th2 cells is of great significance in the treatment of tumours.^60^, ^61^ All these results indicate that upregulation of PPP1R14B expression can inhibit the antitumour immune response in UCEC patients.

To investigate the role of PPP1R14B in predicting the prognosis of patients, we used the TCGA database to analyse the relationship between PPP1R14B expression and clinicopathological features. The expression of PPP1R14B was significantly correlated with histological grade, histological type and tumour invasion. Furthermore, 105 clinical samples were collected, Western blotting and immunohistochemistry were employed to detect the expression of PPP1R14B in UCEC tissues, and the relationships between PPP1R14B and clinical characteristics were analysed. The results showed that PPP1R14B was correlated with FIGO grade and pathological grade (*p* < 0.05). This was consistent with the results of bioinformatics analysis, indicating that high expression of PPP1R14B may indicate poor prognosis of tumour patients. In the following survival analysis, the OS, PFI, and DSS of patients with high PPP1R14B expression were significantly shortened compared with those with low PPP1R14B expression. At the same time, the multivariate Cox regression results were used to construct the nomogram plot as a prediction tool for clinical prognosis, and the accuracy of the model was analysed. As seen from the calibration plot, the actual OS values at 1 year, 3 years, and 5 years were in good agreement with the predicted values. Therefore, the nomogram constructed in this study may become a new and valuable prognostic prediction method.

A large number of previous studies have demonstrated the role of other protein‐coding RNAs and non‐coding RNAs in predicting the occurrence and prognosis of endometrial cancer. L1CAM has been shown to be a promising molecular marker for predicting prognosis in a high‐risk “no specific molecular profile” (NSMP) subgroup of patients with UCEC.^62^ Meanwhile, abnormal expression of non‐coding RNAs such as CCAT2, DLEU1, PVT1, LINC01170, MEG3, and FER1L4 in UCEC has also been proven to be related to the occurrence, development, and prognosis of UCEC. These non‐coding RNAs could also be used as prognostic molecular markers to guide the risk stratification of UCEC patients.^63^ These studies suggest the value and feasibility of PPP1R14B as a potential molecular marker in the diagnosis and prognosis assessment of UCEC. The high expression of PPP1R14B in UCEC predicts the poor prognosis of patients, which can help medical workers to screen out patients suitable for the treatment of fertility function preservation, expect the risk of recurrence of patients with UCEC after treatment, determine which patients need further follow‐up and treatment, develop individualized precise treatment programs, and provide new targets for targeted therapy or immune checkpoint inhibitor therapy in patients with recurrence or metastasis of UCEC.

In conclusion, high expression of PPP1R14B can inhibit the antitumour immune response in UCEC patients and participate in various biological processes, such as tumorigenesis, metastasis, and invasion. Therefore, it may become an independent prognostic risk factor that can be used as a diagnostic and prognostic marker in clinical practice to help doctors make more reasonable treatment plans for patients. However, there was a large difference between the number of normal samples and the number of tumour samples in this study, and further studies with large sample sizes are needed in the future. In addition, the results obtained in this study need more experiments, such as animal experiments and cell experiments, to further verify the mechanism of PPP1R14B in promoting UCEC. Since this study is a retrospective study based on the existing RNA sequencing data in TCGA and GEO databases, prospective studies are needed in the future to reduce the bias caused by retrospective studies.

## AUTHOR CONTRIBUTIONS

**Kang He:** Data curation (equal); writing – original draft (equal). **Taiwei Wang:** Investigation (equal); writing – original draft (equal). **Xuemiao Huang:** Writing – review and editing (equal). **Zhaoyun Yang:** Writing – original draft (equal). **Zeyu Wang:** Writing – original draft (equal). **Shuang Zhang:** Data curation (equal); investigation (equal). **Xin Sui:** Data curation (equal); formal analysis (equal). **Junjie Jiang:** Conceptualization (equal); supervision (equal). **Lijing Zhao:** Conceptualization (equal); supervision (equal).

## FUNDING INFORMATION

This study was supported by a grant from the Jilin Provincial Department of Science and Technology project (grant number: 20210204200YY).

## CONFLICT OF INTEREST STATEMENT

The authors declare that they have no competing interests.

## Data Availability

The datasets generated and/or analysed during the current study are available in the GEO (https://www.ncbi.nlm.nih.gov/geo/), TCGA (https://portal.gdc.cancer.gov), and HPA (https://www.proteinatlas.org/) repositories.

## References

[1] Zhang K , Li H , Yan Y , et al. Identification of key genes and pathways between type I and type II endometrial cancer using bioinformatics analysis. Oncol Lett. 2019;18:2464‐2476. doi:10.3892/ol.2019.10550 31452737 PMC6676660

[2] Siegel RL , Miller KD , Fuchs HE , Jemal A . Cancer statistics, 2021. CA Cancer J Clin. 2021;71:7‐33. doi:10.3322/caac.21654 33433946

[3] Charo LM , Plaxe SC . Recent advances in endometrial cancer: a review of key clinical trials from 2015 to 2019. F1000Res. 2019;8:849. doi:10.12688/f1000research.17408.1 PMC6567288 31231511

[4] Gaber C , Meza R , Ruterbusch JJ , Cote ML . Endometrial cancer trends by race and histology in the USA: projecting the number of new cases from 2015 to 2040. J Racial Ethn Health Disparities. 2016;4:895‐903. 10.1007/s40615-016-0292-2 PMC6917984 27753051

[5] Chen P , Yang Y , Zhang Y , Jiang S , Li X , Wan J . Identification of prognostic immune‐related genes in the tumor microenvironment of endometrial cancer. Aging. 2020;12:3371‐3387. doi:10.18632/aging.102817 32074080 PMC7066904

[6] Xu Q , Ge Q , Zhou Y , et al. MELK promotes endometrial carcinoma progression via activating mTOR signaling pathway. EBioMedicine. 2020;51:102609. doi:10.1016/j.ebiom.2019.102609 31915116 PMC7000338

[7] Lagercrantz J , Carson E , Larsson C , Nordenskjöld M , Weber G . Isolation and characterization of a novel gene close to the human phosphoinositide‐specific phospholipase C beta 3 gene on chromosomal region 11q13. Genomics. 1996;31:380‐384. 8838322 10.1006/geno.1996.0063

[8] Eto M , Karginov A , Brautigan DL . A novel phosphoprotein inhibitor of protein type‐1 phosphatase holoenzymes. Biochemistry. 1999;38:16952‐16957. 10606530 10.1021/bi992030o

[9] Deng JT , Sutherland C , Brautigan DL , Eto M , Walsh MP . Phosphorylation of the myosin phosphatase inhibitors, CPI‐17 and PHI‐1, by integrin‐linked kinase. Biochem J. 2002;367:517‐524. 12144526 10.1042/BJ20020522 PMC1222907

[10] Choy MS , Page R , Peti W . Regulation of protein phosphatase 1 by intrinsically disordered proteins. Biochem Soc Trans. 2012;40:969‐974. 22988849 10.1042/BST20120094 PMC3502941

[11] Ceulemans H , Bollen M . Functional diversity of protein phosphatase‐1, a cellular economizer and reset button. Physiol Rev. 2004;84:1‐39. 14715909 10.1152/physrev.00013.2003

[12] Tountas NA , Brautigan DL . Migration and retraction of endothelial and epithelial cells require PHI‐1, a specific protein‐phosphatase‐1 inhibitor protein. J Cell Sci. 2004;117:5905‐5912. 15522888 10.1242/jcs.01506

[13] Figueiredo J , da Cruz E , Silva OAB , Fardilha M . Protein phosphatase 1 and its complexes in carcinogenesis. Curr Cancer Drug Targets. 2014;14:2‐29. 24200083 10.2174/15680096113136660106

[14] Worley MJ , Liu S , Hua Y , et al. Molecular changes in endometriosis‐associated ovarian clear cell carcinoma. Eur J Cancer. 2015;51:1831‐1842. doi:10.1016/j.ejca.2015.05.011 26059197 PMC4532605

[15] Wang R , Wu Y , Yu J , Yang G , Yi H , Xu B . Plasma messenger RNAs identified through bioinformatics analysis are novel, non‐invasive prostate cancer biomarkers. OncoTargets Ther. 2020;13:541‐548. doi:10.2147/OTT.S221276 PMC6974148 32021296

[16] Zhao M , Shao Y , Xu J , Zhang B , Li C , Gong J . LINC00466 impacts cell proliferation, metastasis and sensitivity to temozolomide of glioma by sponging miR‐137 to regulate PPP1R14B expression. OncoTargets Ther. 2021;14:1147‐1159. doi:10.2147/OTT.S273264 PMC7903952 33642868

[17] Deng M , Peng L , Li J , Liu X , Xia X , Li G . PPP1R14B is a prognostic and immunological biomarker in Pan‐cancer. Front Genet. 2021;12:763561. doi:10.3389/fgene.2021.763561 34858479 PMC8631915

[18] Xiang N , Chen T , Zhao X , Zhao M . In vitro assessment of roles of PPP1R14B in cervical and endometrial cancer. Tissue Cell. 2022;77:101845. doi:10.1016/j.tice.2022.101845 35679681

[19] Vivian J , Rao AA , Nothaft FA , et al. Toil enables reproducible, open source, big biomedical data analyses. Nat Biotechnol. 2017;35:314‐316. doi:10.1038/nbt.3772 28398314 PMC5546205

[20] Day RS , McDade KK , Chandran UR , et al. Identifier mapping performance for integrating transcriptomics and proteomics experimental results. BMC Bioinform. 2011;12:213. doi:10.1186/1471-2105-12-213 PMC3124437 21619611

[21] Day RS , McDade KK . A decision theory paradigm for evaluating identifier mapping and filtering methods using data integration. BMC Bioinform. 2013;14:223. doi:10.1186/1471-2105-14-223 PMC3734162 23855655

[22] Davis S , Meltzer PS . GEOquery: a bridge between the gene expression omnibus (GEO) and BioConductor. Bioinformatics. 2007;23:1846‐1847. 17496320 10.1093/bioinformatics/btm254

[23] Ritchie ME , Phipson B , Wu D , et al. Limma powers differential expression analyses for RNA‐sequencing and microarray studies. Nucleic Acids Res. 2015;43:e47. doi:10.1093/nar/gkv007 25605792 PMC4402510

[24] Hu K , Tian Y , Du Y , et al. Atrazine promotes RM1 prostate cancer cell proliferation by activating STAT3 signaling. Int J Oncol. 2016;48:2166‐2174. doi:10.3892/ijo.2016.3433 26984284

[25] Chen J , Liu J , Wu S , et al. Atrazine promoted epithelial ovarian cancer cells proliferation and metastasis by inducing low dose reactive oxygen species (ROS). Iran J Biotechnol. 2021;19:e2623. doi:10.30498/IJB.2021.2623 34435054 PMC8358173

[26] Chen J , Xia Y , Peng Y , et al. Analysis of the association between KIN17 expression and the clinical features/prognosis of epithelial ovarian cancer, and the effects of KIN17 in SKOV3 cells. Oncol Lett. 2021;21:475. doi:10.3892/ol.2021.12736 33907585 PMC8063336

[27] Love MI , Huber W , Anders S . Moderated estimation of fold change and dispersion for RNA‐seq data with DESeq2. Genome Biol. 2014;15:550. 25516281 10.1186/s13059-014-0550-8 PMC4302049

[28] Jensen LJ , Kuhn M , Stark M , et al. STRING 8‐‐a global view on proteins and their functional interactions in 630 organisms. Nucleic Acids Res. 2009;37:D412‐D416. doi:10.1093/nar/gkn760 18940858 PMC2686466

[29] Yu G , Wang L‐G , Han Y , He Q‐Y . clusterProfiler: an R package for comparing biological themes among gene clusters. Omics. 2012;16:284‐287. doi:10.1089/omi.2011.0118 22455463 PMC3339379

[30] Walter W , Sánchez‐Cabo F , Ricote M . GOplot: an R package for visually combining expression data with functional analysis. Bioinformatics. 2015;31:2912‐2914. doi:10.1093/bioinformatics/btv300 25964631

[31] Subramanian A , Tamayo P , Mootha VK , et al. Gene set enrichment analysis: a knowledge‐based approach for interpreting genome‐wide expression profiles. Proc Natl Acad Sci U S A. 2005;102:15545‐15550. 16199517 10.1073/pnas.0506580102 PMC1239896

[32] Hänzelmann S , Castelo R , Guinney J . GSVA: gene set variation analysis for microarray and RNA‐seq data. BMC Bioinform. 2013;14:7. doi:10.1186/1471-2105-14-7 PMC3618321 23323831

[33] Bindea G , Mlecnik B , Tosolini M , et al. Spatiotemporal dynamics of intratumoral immune cells reveal the immune landscape in human cancer. Immunity. 2013;39:782‐795. doi:10.1016/j.immuni.2013.10.003 24138885

[34] Gu Z , Gu L , Eils R , Schlesner M , Brors B . Circlize implements and enhances circular visualization in R. Bioinformatics. 2014;30:2811‐2812. doi:10.1093/bioinformatics/btu393 24930139

[35] Liu J , Lichtenberg T , Hoadley KA , et al. An integrated TCGA Pan‐cancer clinical data resource to drive high‐quality survival outcome analytics. Cell. 2018;173:400‐416.e11. doi:10.1016/j.cell.2018.02.052 29625055 PMC6066282

[36] Llauradó M , Ruiz A , Majem B , et al. Molecular bases of endometrial cancer: new roles for new actors in the diagnosis and the therapy of the disease. Mol Cell Endocrinol. 2012;358:244‐255. doi:10.1016/j.mce.2011.10.003 22037169

[37] Cavaliere AF , Perelli F , Zaami S , et al. Fertility sparing treatments in endometrial cancer patients: the potential role of the new molecular classification. Int J Mol Sci. 2021;22:12248. doi:10.3390/ijms222212248 34830129 PMC8625356

[38] Rogers MA , Langbein L , Praetzel‐Wunder S , Giehl K . Characterization and expression analysis of the hair keratin associated protein KAP26.1. Br J Dermatol. 2008;159:725‐729. doi:10.1111/j.1365-2133.2008.08743.x 18647308

[39] Homberg M , Magin TM . Beyond expectations: novel insights into epidermal keratin function and regulation. Int Rev Cell Mol Biol. 2014;311:265‐306. doi:10.1016/B978-0-12-800179-0.00007-6 24952920

[40] Karsch S , Büchau F , Magin TM , Janshoff A . An intact keratin network is crucial for mechanical integrity and barrier function in keratinocyte cell sheets. Cell Mol Life Sci. 2020;77:4397‐4411. doi:10.1007/s00018-019-03424-7 31912195 PMC11104923

[41] Bozza WP , Zhang Y , Zhang B . Cytokeratin 8/18 protects breast cancer cell lines from TRAIL‐induced apoptosis. Oncotarget. 2018;9:23264‐23273. doi:10.18632/oncotarget.25297 29796187 PMC5955420

[42] Zhang N , Zhang R , Zou K , et al. Keratin 23 promotes telomerase reverse transcriptase expression and human colorectal cancer growth. Cell Death Dis. 2017;8:e2961. doi:10.1038/cddis.2017.339 28749462 PMC5550880

[43] Bilandzic M , Rainczuk A , Green E , et al. Keratin‐14 (KRT14) positive leader cells mediate mesothelial clearance and invasion by ovarian cancer cells. Cancer. 2019;11:1228. doi:10.3390/cancers11091228 PMC6769856 31443478

[44] Samuels TL , Zimmermann MT , Zeighami A , et al. RNA sequencing reveals cancer‐associated changes in laryngeal cells exposed to non‐acid pepsin. Laryngoscope. 2021;131:121‐129. doi:10.1002/lary.28636 32202667

[45] Tuupanen S , Hänninen UA , Kondelin J , et al. Identification of 33 candidate oncogenes by screening for base‐specific mutations. Br J Cancer. 2014;111:1657‐1662. doi:10.1038/bjc.2014.429 25117815 PMC4200084

[46] Sandgren J , Holm S , Marino AM , et al. Whole exome‐ and mRNA‐sequencing of an AT/RT case reveals few somatic mutations and several deregulated Signalling pathways in the context of SMARCB1 deficiency. Biomed Res Int. 2015;2015:862039. doi:10.1155/2015/862039 26998479 PMC4780067

[47] Lin M , Lei T , Zheng J , Chen S , du L , Xie H . UBE2S mediates tumor progression via SOX6/β‐catenin signaling in endometrial cancer. Int J Biochem Cell Biol. 2019;109:17‐22. doi:10.1016/j.biocel.2019.01.014 30690078

[48] Bateman NW , Teng PN , Hope E , et al. Jupiter microtubule‐associated homolog 1 (JPT1): a predictive and pharmacodynamic biomarker of metformin response in endometrial cancers. Cancer Med. 2020;9:1092‐1103. doi:10.1002/cam4.2729 31808620 PMC6997075

[49] Zheng J , Zhang Y‐W , Pan Z‐F . Dysregulation of MAD2L1/CAMK2A/PTTG1 gene cluster maintains the stemness characteristics of uterine corpus endometrial carcinoma. Zhongguo Yi Xue Ke Xue Yuan Xue Bao. 2021;43:685‐695. doi:10.3881/j.issn.1000-503X.13271 34728029

[50] Liu J , Wan YC , Li S , et al. Identification of aberrantly methylated differentially expressed genes and associated pathways in endometrial cancer using integrated bioinformatic analysis. Cancer Med. 2020;9:3522‐3536. doi:10.1002/cam4.2956 32170852 PMC7221444

[51] Zhang X , Cao G , Diao X , Bai W , Zhang Y , Wang S . Vimentin protein In situ expression predicts less tumor metastasis and overall better survival of endometrial carcinoma. Dis Markers. 2022;2022:5240046. doi:10.1155/2022/5240046 35320951 PMC8938074

[52] Bokhari AA , Baker TM , Dorjbal B , et al. Nestin suppression attenuates invasive potential of endometrial cancer cells by downregulating TGF‐β signaling pathway. Oncotarget. 2016;7:69733‐69748. doi:10.18632/oncotarget.11947 27626172 PMC5342511

[53] Carazo A , Macáková K , Matoušová K , Krčmová LK , Protti M , Mladěnka P . Vitamin a update: forms, sources, kinetics, detection, function, deficiency, therapeutic use and toxicity. Nutrients. 2021;13:1703. doi:10.3390/nu13051703 34069881 PMC8157347

[54] Psilopatis I , Pergaris A , Vrettou K , Tsourouflis G , Theocharis S . The EPH/ephrin system in gynecological cancers: focusing on the roots of carcinogenesis for better patient management. Int J Mol Sci. 2022;23:3249. doi:10.3390/ijms23063249 35328669 PMC8949008

[55] Ben‐Porath I , Thomson MW , Carey VJ , et al. An embryonic stem cell‐like gene expression signature in poorly differentiated aggressive human tumors. Nat Genet. 2008;40:499‐507. doi:10.1038/ng.127 18443585 PMC2912221

[56] Kinsey M , Smith R , Lessnick SL . NR0B1 is required for the oncogenic phenotype mediated by EWS/FLI in Ewing's sarcoma. Mol Cancer Res. 2006;4:851‐859. 17114343 10.1158/1541-7786.MCR-06-0090

[57] Meissner A , Mikkelsen TS , Gu H , et al. Genome‐scale DNA methylation maps of pluripotent and differentiated cells. Nature. 2008;454:766‐770. doi:10.1038/nature07107 18600261 PMC2896277

[58] Buisseret L , Pommey S , Allard B , et al. Clinical significance of CD73 in triple‐negative breast cancer: multiplex analysis of a phase III clinical trial. Ann Oncol. 2018;29:1056‐1062. doi:10.1093/annonc/mdx730 29145561 PMC5913595

[59] Chen H , Xie J , Jin P . Assessment of hazard immune‐related genes and tumor immune infiltrations in renal cell carcinoma. Am J Transl Res. 2020;12:7096‐7113. 33312353 PMC7724327

[60] Sharma A , Rajappa M , Saxena A , Sharma M . Cytokine profile in Indian women with cervical intraepithelial neoplasia and cancer cervix. Int J Gynecol Cancer. 2007;17:879‐885. 17343606 10.1111/j.1525-1438.2007.00883.x

[61] Johnson SD , De Costa A‐MA , Young MRI . Effect of the premalignant and tumor microenvironment on immune cell cytokine production in head and neck cancer. Cancer. 2014;6:756‐770. doi:10.3390/cancers6020756 PMC4074802 24698959

[62] Ravaggi A , Capoferri D , Ardighieri L , et al. Integrated biomarker analysis reveals L1CAM as a potential stratification marker for No specific molecular profile high‐risk endometrial carcinoma. Cancer. 2022;14:5429. doi:10.3390/cancers14215429 PMC9658459 36358847

[63] Piergentili R , Zaami S , Cavaliere AF , et al. Non‐coding RNAs as prognostic markers for endometrial cancer. Int J Mol Sci. 2021;22:3151. doi:10.3390/ijms22063151 33808791 PMC8003471

